# The fidelity and dose of message delivery on infant and young child feeding practice and nutrition sensitive agriculture in Ethiopia: a qualitative study from the Sustainable Undernutrition Reduction in Ethiopia (SURE) programme

**DOI:** 10.1186/s41043-019-0187-z

**Published:** 2019-10-21

**Authors:** Mihretab M. Salasibew, Cami Moss, Girmay Ayana, Desalegn Kuche, Solomon Eshetu, Alan D. Dangour

**Affiliations:** 1 London School of Hygiene and Tropical Medicine, London, UK; 2 Ethiopian Public Health Institute, Addis Ababa, Ethiopia

**Keywords:** Infant and young child feeding, Nutrition-sensitive agriculture, Exclusive breastfeeding, Complementary feeding, Dietary diversity, Behaviour change communication

## Abstract

**Background:**

In Ethiopia, 38% of children under 5 years of age are stunted (low height for age). A novel government-led intervention called the Sustainable Undernutrition Reduction in Ethiopia (SURE) aims to tackle the burden of stunting by improving complementary feeding and dietary diversity among young children. The SURE programme design applies a transtheoretical model of behaviour change, whereby exposure to recommended infant and young child feeding (IYCF) and nutrition-sensitive agriculture messages is a first stage to adopting key behaviours. This qualitative study explored the fidelity and dose of the IYCF and nutrition-sensitive agriculture messages delivered by extension workers.

**Methods:**

A qualitative study was conducted across four regions in Ethiopia (Oromiya, Amhara, SNNP and Tigray) between April and October 2017. Across the four regions, 81 key informant interviews, 90 FGDs and 81 observations were conducted with 180 extension workers, 18 development agents and 54 mother-father pairs. Digitally recorded audio files were transcribed verbatim, and the data were analysed based on a framework analysis approach using NVivo (version 12) by coding and categorising texts into major themes and sub-themes.

**Results:**

SURE target households had the intended exposure to messages about exclusive breastfeeding, timing of initiation of complementary feeding, food groups, diversified food consumption, irrigation, rearing small animals and vegetables. Few households reported receiving messages on the content or frequency of complementary feeding of a child beyond 6 months of age. Frequency of household visits and hence exposure to SURE messages was also variable. Agricultural messages delivered during household visits focussed on improving standard agricultural practices and rarely covered the importance of nutrition-sensitive agriculture to improve household or child nutrition.

**Conclusion:**

Despite variability observed in the breadth and depth of messages delivered, large-scale behaviour change communication programmes can achieve moderate to good message exposure among target groups. Qualitative data provide an in-depth insight into fidelity and may supplement our understanding of programme roll-out and implementation. Further research is required to understand longer-term message saturation including frequency and reach.

## Background

Globally, undernutrition remains a major public health problem with 155 million stunted (low height for age) under-five children. Africa and Asia carry the heaviest burden of undernutrition, with 59 million and 87 million under 5 years of age children stunted, respectively [1]. In Ethiopia, more than a third of under-five children are still stunted (despite a substantial reduction from 58% in 2000 to 38% in 2016 [2]); 10% are wasted (low weight for height) and 24% are underweight (low weight for age). While breastfeeding is nearly universal in Ethiopia and 73% of all newborns initiate breastfeeding within an hour of birth, only 58% of infants are exclusively breastfed up to 6 months of age and only 7% and 14% of children under 24 months, respectively, receive the minimum acceptable diet and consume diverse diets [2].

To support child growth and development in the first 1000 days (the period from conception until a child’s second birthday) [3–8], a list of messages delivered by the health sector to improve infant and young child feeding (IYCF) practices has been developed [9]. The important role of other sectors (agriculture, water and sanitation, education, etc.) in addressing undernutrition has been highlighted [10], but similar evidence-based nutrition sensitive agriculture messages have not so far been produced.

The Sustainable Undernutrition Reduction in Ethiopia (SURE) programme (2016–2019) is the first government-led inter-sectoral programme aiming to join the health and agriculture sectors to improve complementary feeding practices and consumption of diversified diet in Ethiopia. Health and agriculture extension workers jointly visit households every 2 months to provide IYCF counselling and nutrition-sensitive agriculture advice to mothers and fathers of children under 24 months, inclusive of pregnant women and fathers-to-be [11]. Health and agriculture development agents (women or men, respectively, selected from one in each six households to support local government administration) further support message delivery during routine weekly meetings with women or men from the remaining five households in the network.

The programme is being implemented in four major regions in Ethiopia namely: Oromiya, Amhara, SNNP and Tigray. In 2017, over 7000 health and agriculture extension workers received SURE training [12]. Training included the recommended list of IYCF messages for mothers of children under 2 years of age [9] as well as nutrition-sensitive agriculture messages developed locally for the SURE programme (Table 1) in partnership with the federal Ministries of Health and Agriculture and Natural Resources in Ethiopia.

**Table 1:** SURE nutrition-sensitive agriculture messages for care givers of healthy under-two children

Selection and diversity of crops	
Provide nutritious foods for your family with poultry, small livestock, vegetable gardening	
Plant different crops to be harvested at different times of year	
Grow plants that live for more than 1 year, which is useful for food security	
Grow a variety of cereals whenever possible. Intercrop with legumes	
Grow diverse foods such as vegetables and fruits to eat and to sell	
Attend a farmer demonstration centre or talk to your agriculture extension agent for help with crop selection	
Use improved seed varieties	
Consider producing and eating nutritious foods in your area that are available but not commonly consumed, such as wild fruits	
Land management	
Rotate crops among different fields	
Practice intercropping (rows of legumes and/or vegetables with main staples)	
Practice agroforestry (planting trees or shrubs to reduce erosion) in or around planting fields	
Practice conservation farming and minimum tillage methods to reduce soil erosion, such as terracing	
Use drainage methods to prevent excessive soil water logging or run off	
Plough manure from livestock back into the soil to fertilise it	
Water management	
Keep animals away from water sources	
Use improved latrines, do not practice open defecation	
Filter or use settling ponds to improve water quality	
Harvest water during the rainy season	
Livestock	
Raise poultry, goats or sheep, or larger animals, especially high yielding or improved varieties	
Use confined/caged poultry production systems	
Attend livestock demonstrations to learn how to care for livestock and keep them healthy	
Keep livestock out of the house to avoid infectious disease	
Keep eggs and milk for consumption, especially by children	
Income from agriculture	
Fathers and mothers allocate some money to buy nutritious foods at the market	
Fathers and mothers save money to use for nutrition or for your child’s treatment when sick	
Mothers participate in agriculture or livestock to generate income for the family	
Fathers empower mothers to budget money for the health and nutrition of the family	
Shared role of fathers and mothers on their family nutrition	
Fathers help your wife with household tasks, so she can ensure your child’s good diet	
Fathers and mothers play with your child to promote healthy growth of mind and body	
Fathers and mothers work together with respect and partnership to help your children grow well	
Food handling, processing and storage	
Use safe food preparation and storage behaviours	
Use good pre- and post-harvest storage and handling practices	
Use proper storage for vegetables and diffused light storage for seeds and potatoes	

The SURE programme evaluation, which is being conducted in a sample of 36 intervention and 36 comparison districts, aims to evaluate the impact of the SURE programme on stunting and the minimum acceptable diet in children under 5 years of age. The evaluation consists of impact evaluation, process evaluation and cost-effectiveness analysis [11]. This qualitative study, part of the process evaluation series, explored the fidelity (the quality of what was delivered) and the dose (the quantity of what was delivered) of IYCF and nutrition-sensitive agriculture message delivery. Assessment of fidelity and dose of programme implementation supports understanding of how a multi-component intervention produces its effects [13] (new ref), informs the government of how well activities have been delivered [14] (new ref) and, consequently, measures that might be taken for course correction [15] (new ref).

## Methods

### Theoretical framework

The SURE programme design adopted the transtheoretical model of behaviour change that defines a series of stages that individuals pass through before adopting and maintaining a behaviour [16, 17]. Exposure to recommended IYCF and nutrition-sensitive agriculture messages is a first stage to adopting key behaviours that in turn lead to improved complementary feeding practices and diversified food consumption. The theory has been critiqued since people who were actively making a change (actors) or maintaining a change (maintainers) had done so for a range of different periods of time, with no evident stages or cut-off points [18], but the theory plays a key role in the design of many behavioural change interventions, including those in the health, agriculture and nutrition sectors [19–22].

There are five stages in achieving behaviour change according to the transtheoretical model [16, 17]: precontemplation, contemplation, action, maintenance and termination. In this study, we investigated the first two stages of transtheoretical model (precontemplation and contemplation) to understand fidelity and dose of infant and young child feeding and nutrition-sensitive agriculture message delivery.

### Study design

This was a qualitative study designed to elicit in-depth understanding of what and how much of the recommended IYCF and nutrition-sensitive agriculture messages were delivered. Methods and findings from this study are reported as per the consolidated criteria for reporting qualitative research (COREQ) [23].

### Study population

Eligible study participants included extension workers—trained to provide counselling—and household members, who received IYCF counselling and nutrition-sensitive agriculture advice in the 36 SURE intervention sample districts across four regions of Ethiopia (Oromiya, Amhara, SNNP and Tigray).

### Sample selection

In Ethiopia, administrative structures are designed as federal, regional, zonal, district (woreda) and sub-district (kebele) [24]. From the list of 36 districts selected for the SURE programme evaluation [11], we purposively selected 18 districts for this study: six districts in Oromiya region (which had a relatively greater number of districts receiving the SURE programme) and four districts in each of Tigray, Amhara and SNNP regions. We selected no more than one district per zone (the administrative unit smaller that region). From each district, we randomly selected six kebeles (smallest administrative unit). The final sample was 108 kebeles in 18 districts across four regions.

Study participants were purposively selected to participate in two rounds of data collection in line with the phased roll-out of specific programme components. In the first round (April–May, 2017), a pair of health and agriculture extension workers were selected from all 18 districts. A single health or agriculture development agent was selected across the 18 districts. In the second round (September–October, 2017), all participants were selected from kebeles with five pairs of health and agriculture extension workers selected from nine kebeles. Three mother-father pairs were selected from nine kebeles. Extension workers and development agents were invited to participate in the study based on their availability on the date and time of the interview. Mother-father pairs were eligible for interviews if their household was receiving SURE intervention, and they had under-two children living in the household (Table 2).

**Table 2:** Participants of the study

Study population	1st round (April 2017)	2nd round (October 2017)			Total
	KIIs	KIIs	FGDs	Observations	
Health extension workers^1^	18	0	45	27	90
Agriculture extension workers^2^	18	0	45	27	90
Health development agents^3^	9	0	0	0	9
Agriculture development agents^4^	9	0	0	0	9
Mothers and fathers	0	27	0	27	54
Total	54	27	90	81	

*KIIs* key informant interviews, *FGDs* focus group discussions

^1^Health extension workers: Trained female health workers who deliver a package of health services to the community

^2^Agriculture extension workers: Trained male agriculture workers who deliver a package of agriculture services to the community

^3^Health development agent: Volunteer community health agents who support health extension workers

^4^Agriculture development agent: Volunteer community agriculture agents who support agriculture extension workers

### Data collection

Programme activities were introduced in phases, and there were two rounds of data collection aligned with the phased roll out. In the first round of data collection, key informant interviews were conducted and in the second round of data collection, key informant interviews, focus group discussions and observations were conducted.

Health and agricultural extension workers were interviewed using a topic guide (see Additional files 1, 2 and 3) and participated in focus group discussions (see Additional files 5 and 6) about their experiences of SURE training as well as the knowledge and skills they reportedly gained. We observed household visits conducted jointly by health and agriculture workers using an observation form (see Additional file 7). Mother-father pairs, who were recipients of the SURE intervention, were interviewed about IYCF and nutrition-sensitive agriculture messages using a topic guide (see Additional file 4).

Ten enumerators and two regional supervisors were involved in this study, and they received a 5-day training on the SURE programme and its components including overview of the programme components, principles of qualitative studies and key skills required to conduct key informant interviews, focus group discussions and non-participant observations. Prior to data collection, the tools (topic guide, observation form, informed consent form and demographic characteristics of respondents’ forms) were pre-tested in a day-long field trip to Mareko Woreda in SNNP, 150 km from Addis Ababa.

During the field test, enumerators interviewed health and agriculture extension workers, health and agriculture development agents and mother or fathers of households visited for counselling; conducted focus group discussions separately with health extension workers and agriculture extension workers; and observed joint house to house counselling. Revisions were made following the field test including correction in the wording of the translation, removing duplicates and rewording the consent form. Enumerators received further training on probing techniques and verbatim transcription.

Key informant interviews and focus group discussions were held in Amharic language in Amhara and SNNP regions, in Tigrigna in Tigray region and Oromifa in Oromiya region. Interviews and focus group discussions took on average 40 min and 1 h, respectively, and were recorded using a digital audio recorder upon receiving consent. Audio recordings were kept in a password protected folder in the enumerators’ laptop computers and were transferred to the Ethiopian Public Health Institute (EPHI) at the earliest possible time via secure email to the principal investigator—the only person who had access to the data at EPHI. Audio recordings were transcribed verbatim, and observation notes were typed and compiled in a Microsoft word document.

### Data analysis

**Fig. 1:**
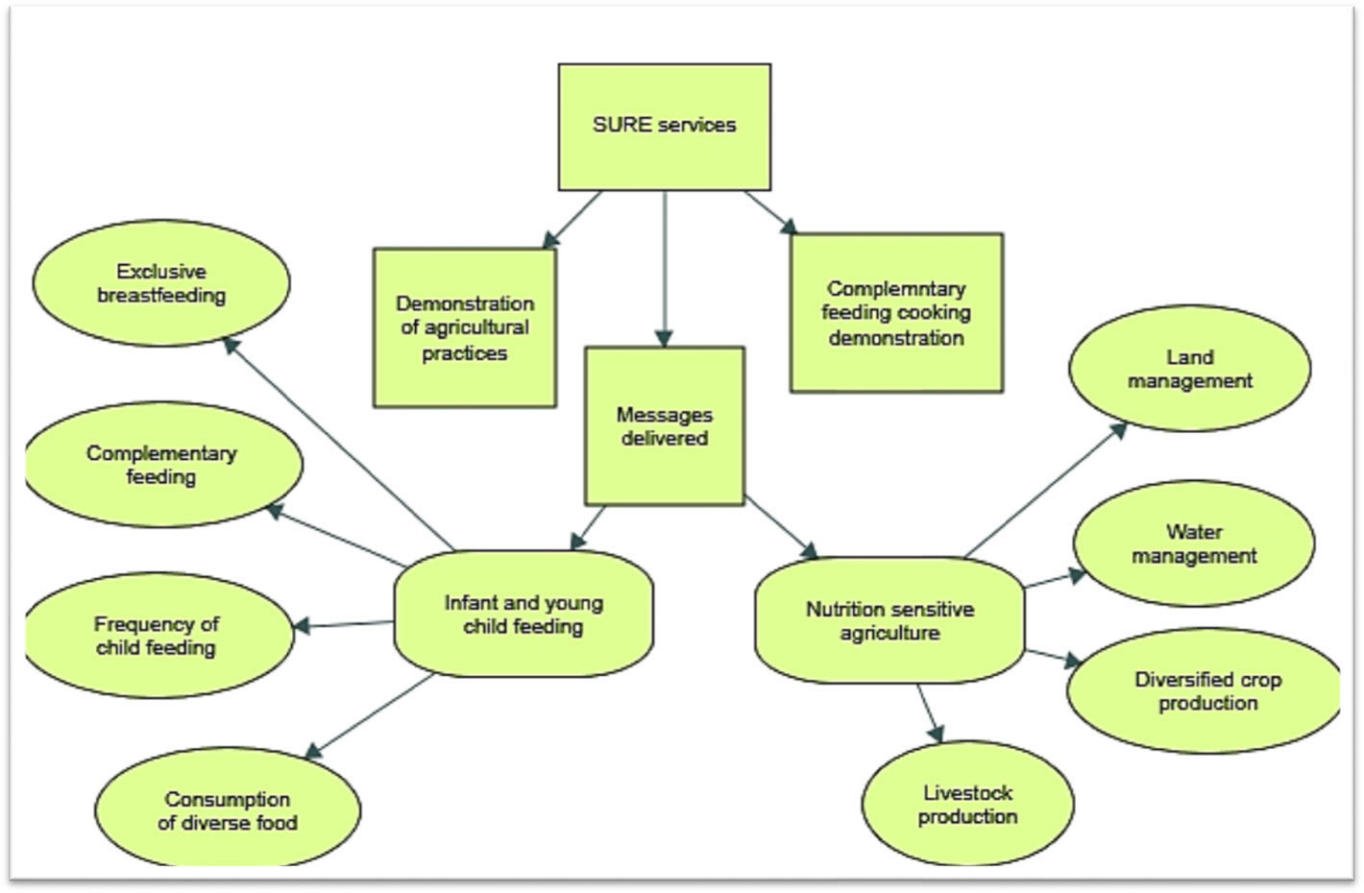
SURE service delivery themes and sub-themes

A framework analysis approach was used to analyse the collected qualitative data. Framework analysis provides a transparent approach by using systematic and visible stages as to how the results or conclusions were made using the data [25]. Deductive and inductive approaches were used to code contents of a transcript. In the deductive approach, contents were coded by pre-determined themes based on the topic guide and research questions, whereas in the inductive approach, contents were coded by creating new emerging themes. Identified themes were categorised into broader themes, i.e. SURE services delivered, service delivery approaches and multisectoral coordination for nutrition. SURE services was further categorised into three sub-themes: messages delivered, demonstration of agricultural practices and complementary feeding cooking demonstration. We then charted and mapped specific codes, using the ‘maps’ function of the Nvivo software version 12, into these sub-themes (Fig. 1).

## Results

Across the four regions, 81 key informant interviews, 90 FGDs and 81 observations were conducted with 180 extension workers, 18 development agents and 54 mother-father pairs (Table 2). Health and agriculture extension workers received training on various topics covering IYCF and nutrition sensitive agriculture (Table 1). Thematic analysis of the contents of the training and messages delivered indicate that the training focused more on knowledge than on facilitation skills required for household visits, including age-appropriate counselling and practical demonstrations.

Findings are presented in two sub-themes: IYCF and nutrition-sensitive agriculture message delivery (Fig. 1).

### IYCF messages delivered

Mothers of children under 24 months, who were recipients of SURE services, reported receiving infant and young child feeding messages delivered by trained extension workers. There was a strong emphasis on exclusive breastfeeding messages and timing of initiation of complementary feeding.

She (health extension worker) advised me to feed only breast milk from birth up to six months. In addition, she taught us the child needs supplementary food after six months… (Mother, SNNP region)

They (extension workers) told us to feed only breastmilk up to his six months age. Complementary foods that includes egg and milk should start when the child is six months old and we are feeding our kids like this. (Mother, Amhara region)

We observed that messaging was at times incorrectly aligned with the age of the child at the time of the visit, for example, breastfeeding techniques and demonstrations (a topic for which health extension workers are already well-drilled) were frequently overemphasised for children over 6 months of age, and complementary feeding messages lacked specificity. In interviews and FGDs, messages about early initiation of breastfeeding within an hour after birth and the need to feed colostrum to a newborn baby were rarely mentioned. Introduction of complementary food after 6 months of age was frequently mentioned but few (6 out of 27 households) could accurately describe the age-appropriate meals (by child age group) or how often a child should be fed (frequency).

The mother had 11 months old child at the time of the joint household visit. The health extension worker delivered messages on appropriate positioning during breastfeeding, latching/attachment, active feeding, complementary feeding and family planning... (Observation, SNNP region)

The workers began their counselling by greeting the mother who was with her newborn baby. They taught the mother about the importance of breastfeeding for newborn baby, how to keep the hygiene during breastfeeding and the need to allow the baby complete suckling a breast before moving to the other. The workers continued advising the mother about the introduction of complimentary food with semi-solid porridge and feeding snacks between meals when the child is six months old… (Observation, Oromiya region)

…the health extension workers told us how many times we should feed our children…we have now changed, and a child should receive complementary feeding three times a day together with breastmilk… (Mother, Amhara region)

…they (health extension workers) told me to feed three times a day, morning, day time, and evening… (Mother, Tigray region)

… for a child aged 6 months to 11 months, we have to give at least 1 to 2 coffee cups of complementary food per day… (Mother, SNNP region)

Dietary messages about diversified food consumption were consistently mentioned by caregivers but were rarely accompanied by discussions of the food groups or minimum consumption recommendations.

…to properly feed our children, they advised us to give the child porridge made of legumes, pea, potato, and spinach ... They instructed us to prepare porridge by cooking with the residual water of the spinach… (Mother, Amhara region)

They (health extension workers) told us about how to feed different foods for our children after 6 months …such as vegetables, chicken, egg, potato, anchote, wheat, teff, sorghum and maize (Mother, Oromiya region)

The need to keep good personal hygiene, including hand washing, was reportedly emphasised by extension workers and development agents while advising mothers on complementary food preparation.

They (Health extension workers) advised us to give child care, maintain the child personal, house as well as food hygiene … (Mother, Amhara region)

They (Health extension workers) tell us about hand washing before cooking food, washing after cooking food, washing children before feeding, washing children after feeding, washing our hands before feeding children and so on... (Mother, SNNP region)

… during 1-5 community network discussions, I teach mothers on how to keep their hygiene and how to feed their children from different types of foods…. (Health development agent, SNNP region)

Household reports of infant and young child feeding messages were in line to those reported delivered by extension workers health and agriculture workers (Table 3).

**Table 3:** Examples of IYCF messages quoted from interviews with extension workers and health and agriculture workers

Exclusive breastfeeding	Complementary feeding		
	Initiation of complementary feeding	Quantity/frequency of feeding	Diversity of complementary foods
‘Child should get only breast milk until 6 months…’ (HDA, Oromiya)	‘At the age of 6 months, child should start to get additional food…’ (ADA, Amhara)	‘After six months, the baby should get additional food at least three to four times a day and breast feeding should continue…’ (AEW, Tigray)	‘There are twelve different food items that we can get from production…’ (ADA, Oromiya)
‘A child from birth up to 6 months should only feed breast milk…’ (AEW, SNNP)	‘Prepare and give semi-solid porridge after 6 months…’ (ADA, SNNP)	‘… So, the child should get more than two spoons full during feeding’ (AEW, Tigray)	‘…feed children different food types in the form of porridge’ (HEW, SNNP)
‘Child from birth to 6 months should get only mother’s breast milk…’ (AEW, Oromiya)	‘After six months, the child should also get complementary food…’ (AEW, Tigray)	‘From 6 to 9 months, children should get two to three cups additional food. From 6 to 12 months, they should get three cups. From 12 to 24 months, they can have more complementary food…’ (AEW, Oromiya)	‘All mother and father should be aware of not to grow any child by eating only Shiro (stew made from peas)…. for example, if the child eats Shiro now, then he has to eat vegetable for the next meal interchangeably’ (ADA, Amhara)
‘Baby should not take any additional food including water in the first six months…’ (AEW, Tigray)	‘From 6 to 12 month, start to give small foods. Small foods mean for example, porridge…’ (AEW, SNNP)	‘From 6 to 9 months, two coffee cups of porridge and from 9 to 23 months, four coffee cups…’ (AEW, SNNP)	‘If we give children fruits, they will have healthy growth, healthy brain, good in learning … we have banana, we have avocado, we have oats. we have all kind of fruits in our land. But, it’s because of lack of knowledge that our children lagged …we learnt to feed different foods…’ (ADA, SNNP)
‘For a child up to 6 months, she should only breastfeed…’ (AEW, Amhara)	‘After six months, they should give additional food in parallel to breast milk…’ (HEW, Oromiya)	‘For a child 6–11 month, we should give at least 1 to 2 coffee cups of additional food per day…’ (HEW, SNNP)	‘A child should get porridge containing different food groups like dried and refined meat…’ (AEW, Tigray)
‘From birth up to six months, the new baby should also be given breast milk only, not even water…’ (HEW^3^, Tigray)			
‘Child up to 6 months only mothers breast milk…’ (HEW, Amhara)			

*HDA* health development agents, *AEW* agriculture extension worker, *HEW* health extension worker, *ADA* agriculture development agent

### Nutrition-sensitive agriculture messages delivered

We observed during visits that agricultural messaging was often based on common agricultural techniques for which extension workers were previously well trained, with less emphasis given to nutrition-related production considerations or to the farming or environmental situation specific to a given household.

However, there was a strong emphasis on delivering messages about crops, particularly on diversifying crop production, followed by methods of land preparation and management and irrigation.

…they (agriculture extension workers) told us to plant maize, legume, and soya beans together to increase production and productivity. (Father, Amhara region)

In the past, we didn’t know sowing seeds line by line. Health and agricultural workers in our kebele taught us how to do this... I like the experience I gained through them. (Mother, Oromia region)

…the agricultural extension worker advised farmers on crop rotation and fertilizers …used job aids with pictures and engaged farmers by asking question…. (Observation, Amhara region)

… He [agricultural extension worker] focused on advising farmers about irrigation and didn’t use the food groups poster [part of the program job aid with a pictorial demonstration of food groups] to advise on consumption of diversified food. (Observation, SNNP region)

… we used to produce crops once per year. But after they (agriculture extension workers) started teaching us, we produced different types of crops every 2 or 3 months. For example, we produce carrot within 3 months and followed by tomato …and so on. (Father, SNNPR region)

… to protect the land and improve productivity, I was advised to make terracing, ploughing the farm again and again, using fertilizers, manure or compost to increase productivity. (Father, Tigray region)

I have a plan to produce vegetables using irrigation. If they (agriculture extension workers) continue to support us, we will not go back to the past. Of course, the production of these vegetables is vital for us because we do not need to go to the market to buy these products. (Father, Amhara region)

Households consistently mentioned receiving advisory messages on the need to produce chickens, sheep, goats and other animals for food consumption. Some beneficiaries were able to identify the links between increasing production of animal source foods and good child feeding practices.

… they (agriculture extension workers) told us to change local chickens to American chickens. … the American chicken gives us more milk. The locals give us one and half litre but the American gives us four litres enough to feed my children. (Father, Tigray region)

The shared role of fathers and mothers to produce nutritious foods and ensure good infant and child feeding was also highlighted by some respondents who received SURE programme counselling.

…we both (mother and father) share the responsibility of looking after seeds in the farm as well as feeding our children and sending them to school. (Father, Amhara region)

… my husband help me in caring for the children … changing their clothes. He also looks after the livestock and our farm in order to feed the family. (Mother, Oromia region)

Household reports of nutrition-sensitive agriculture messages were in line to those reportedly delivered by extension workers and agriculture workers (Table 4).

**Table 4:** Examples of nutrition sensitive agriculture messages quoted from interviews with agriculture extension and development agents

Land management	Diversified crops	Livestock	Water management
‘To improve my agricultural production, I should work by making terracing…using fertilizers, manure or compost….’ (ADA, Tigray)	‘Instead of producing only maize, they can also produce beans, peas and such things for diversification…’ (AEW, SNNP)	‘Rearing rudimentary animals like sheep and goats, modern honey bee, and growing special varieties of grasses for animals…’ (ADA, Tigray)	‘Harvest water from rain…’ (AEW, Oromiya)
‘We discuss about how to sow line by line…. then how to plant in line. Secondly…how to remove weed and apply chemical (insecticide)….’ (ADA, Oromiya)	‘Growing variety of fruits and vegetables in their home garden, which are good sources of vitamin A…’ (ADA, Tigray)	‘During our 1 to 5 meeting, we teach that animal and human should be separated. Even chickens should be separated…’ (ADA, SNNP)	‘…they can collect used water in the house and they can use that to produce cabbage’ (AEW, SNNP)
‘…we teach about sowing line by line…’ (ADA, Amhara)	‘We advise them to sow different types of diversified vegetables…different types of fruits …’ (ADA, Amhara)	‘On a small land, they can produce chicken, sheep and goat…’ (AEW, Oromiya)	
‘…sow different vegetables seeds 25 cm apart and by interchanging their soil’ (AEW, Tigray)			
‘… we advise them on how much fertilizer and seeds they need per hectare’ (AEW, SNNP)			

*ADA* agriculture development agent, *AEW* agriculture extension worker

### Frequency of exposure to messages

Following the initial joint programme training, health and agriculture workers reported that they began to visit SURE target households to deliver messages on IYCF and nutrition sensitive agriculture. The number of times households were exposed to these messages was not reported to be consistent and varied from twice a week to once every 2 months.

…we had training and then conducted joint household visits every 2 months together with the health extension workers. (Agriculture extension worker, Amhara region)

We work collaboratively with the health extension workers every week… we visit in every two month to deliver the three nutrition components with the support from health extension workers using the materials we have. (Agriculture extension worker, Amhara region)

It’s every month that we conduct joint household visit. Every month that we conduct joint house hold visit. (Agriculture extension worker, Oromia region)

… we visit households every two weeks or every month because the village is too wide to cover. (Agriculture extension worker, SNNP region)

We observed extension workers give 1 to 2 months of appointments to households for their next visits, and this was consistent with household reports of exposure to the messages. However, it was unclear whether the same messages were repeated in subsequent visits to reinforce an identified behaviour or practice.

… they (health extension workers) come and teach us every month and sometimes every two months. (Mother, Tigray region)

… they (health and agriculture extension workers) jointly come to teach us every two or three months about the nutrition led agriculture and proper use of agricultural products for normal growth of children. (Mother, Amhara region)

At the end of the visit, the extension workers and the mother agreed on an appointment for next month. (Household visit observation, Tigray region)

The extension workers agreed on an action plan with the mother and gave her a follow up appointment in two months time. (Household visit observation, SNNP region)

Both extension workers asked the mother about the action plan agreed in previous visits and the mother told them about what she had been doing during the last two months, particularly on her child feeding practice. (Household visit observation, Amhara region)

## Discussion

In this qualitative study, we collected evidence on fidelity and dose of IYCF and nutrition-sensitive agriculture message delivery in selected SURE districts in Ethiopia. SURE target households had exposure to key messages including exclusive breastfeeding, timing of initiation of complementary feeding, food groups, diversified food consumption, irrigation and rearing small animals and vegetables—consistent with the pre-contemplation and contemplation stages of the transtheoretical theory of behaviour change. Few households reported receiving messages on the composition or frequency of complementary feeding of a child beyond 6 months of age—a key focus of the SURE program. Agricultural messages delivered during household visits were often observed to focus on the improvement of generic agricultural practices and less often focused on identifying practicable solutions to improve household nutrition. Frequency of household visits and hence exposure to SURE messages (dose) was variable.

Previous studies in Ethiopia showed that it is possible to deliver quality IYCF interventions at scale, while creating new knowledge, tools and approaches that can be adapted by others [26]. Similarly, multifunctional community health agents were able to deliver breastfeeding counselling in Brazil at scale within a routine health service and this was associated with a significant increase in rates of exclusive breastfeeding [27]. As reported in our study, the SURE programme was delivered at scale and targeted households were exposed to key IYCF and nutrition-sensitive agriculture messages despite some identified gaps.

Community workers play an important role in delivering nutritional messages to mothers or caregivers [28], and effective training of these workers has been shown to improve feeding frequency, energy intake and dietary diversity of children aged 6 months to 2 years [29]. Consistent with a study in Ethiopia [30], we identified gaps in the delivery of complementary feeding messages. This demonstrated that training alone did not result in sufficient application of counselling skills and it requires rational task allocation, substantial follow-up and recognition of extension workers [26]. In-service nutrition training also improves retention of the necessary knowledge and skills required to manage undernutrition [31, 32]. Other studies [27, 33] suggested trainings with more focus on processes of message delivery than contents.

Few agriculture workers discussed the link between good agricultural practice and nutrition. Increased agricultural production does not necessarily lead to consumption. A similar qualitative study in Ethiopia showed that perceived responsibility of agricultural extension workers was to advise households to improve productivity of cattle and using improved breeds. It was assumed that increasing the availability of milk and meat would automatically result in improved household consumption [34], emphasising the nascent stage of nutrition ownership among agricultural extension workers.

One of the main strengths of this study is that the qualitative study design facilitated in-depth understanding of the views and experiences of study participants, which would not have been possible to capture through survey questionnaires. Furthermore, we managed to recruit a large sample of participants across all regions, both from the beneficiaries of the programme and community workers providing the services. We also triangulated findings through interviews, observations and focus group discussions.

However, there were some limitations in this study. Although we ensured heterogeneity by involving different sets of people with similar characteristics, it may be possible that individuals with similar characteristics may still have different or opposing views or experiences of fidelity in implementation. We found evidence of repeated household visits, but we were unable to determine in full whether households were exposed to the same messages during subsequent visits (i.e. quantity of messages delivered or dose).

Quality of implementation can differ widely across different contexts, and rigorous programme monitoring is recommended to track fidelity [35]. Future studies assessing dose of interventions could collect quantitative data, through mid-term coverage surveys, to complement qualitative descriptions of programme implementation.

## Conclusion

Despite variability observed in the breadth and depth of messages delivered, large-scale behaviour change communication programmes can achieve moderate to good message exposure among target groups. Qualitative data provide an in-depth insight of fidelity and could supplement our understanding of programme roll out and implementation. Further research is required to understand longer-term message saturation including frequency and reach.

## Supplementary information

**Additional file 1.** Topic guide for key informant interviews with health extension workers

**Additional file 2.** Topic guide for key informant interviews with agriculture extension workers

**Additional file 3.** Topic guide for key informant interviews with health and agriculture development agents

**Additional file 4.** Topic guide for key informant interviews with mother-father pairs in a household

**Additional file 5.** Topic guide for focus group discussions with health extension workers

**Additional file 6.** Topic guide for focus group discussions with agriculture extension workers.

**Additional file 7.** Observation note-taking form

## Data Availability

The data that support the findings of this study are available from the Ethiopian Public Health Institute (EPHI), but restrictions apply to the availability of these data, which were used under licence for the current study and so are not publicly available. Data are however available from the authors upon reasonable request and with permission of Solomon Eshetu, Act. Director of Nutrition and Food Science Directorate, EPHI, Ethiopia.
