# Supplementary material for: The fidelity and dose of message delivery on infant and young child feeding practice and nutrition sensitive agriculture in Ethiopia: a qualitative study from the Sustainable Undernutrition Reduction in Ethiopia (SURE) programme
Source: J Health Popul Nutr. 2019 Oct 21;38:29. doi: 10.1186/s41043-019-0187-z (PMC6805331; doi:10.1186/s41043-019-0187-z)
Supplement: Supplementary file 1 — Additional file 1. Topic guide for key informant interviews with health extension workers [file 41043_2019_187_MOESM1_ESM.docx]

## Additional file 1: Topic guide for key informant interviews with health extension workers

1. What are your overall impressions of the SURE training?

Probes:

- - Demonstrations
  - Practice

1. Following the training, what do you understand about infant and young child feeding practices?
2. Following the training, what do you understand about agriculture for nutrition practices?
3. What do you understand about the 3As counselling process for child feeding or agriculture?

Probes:

- 3As steps (assessment, analysis, action)
- Discuss what she/he understand by each component in A’s

1. What do you understand about the men’s and women’s group dialogues?

Probes:

- 7 organizing steps
- Facilitation skills (listening and learning, confidence building)
- Topic discussed

1. What do you understand about your role and responsibilities to implement the SURE programme?

Probes:

- Your role is vis-à-vis the other sector
- Household counselling visits
- Cooking demonstrations
- Gardening demonstrations
- Frequency of services

1. What do you understand about how to use the SURE tools (job aids, pocket guide, seasonal food calendar?)

Probes:

- Who
- When/frequency

1. What do you understand about how to use the SURE monitoring forms?

Probes:

- - Who
  - When/frequency
