# Supplementary material for: The fidelity and dose of message delivery on infant and young child feeding practice and nutrition sensitive agriculture in Ethiopia: a qualitative study from the Sustainable Undernutrition Reduction in Ethiopia (SURE) programme
Source: J Health Popul Nutr. 2019 Oct 21;38:29. doi: 10.1186/s41043-019-0187-z (PMC6805331; doi:10.1186/s41043-019-0187-z)
Supplement: Supplementary file 2 — Additional file 2. Topic guide for key informant interviews with agriculture extension workers [file 41043_2019_187_MOESM2_ESM.docx]

## Additional file 2: Topic guide for key informant interviews with agriculture extension workers

1. Are you aware of the SURE programme? If yes, how did you become aware?

Probes:

- - Briefing
  - On the job training

1. What do you understand about infant and young child feeding practices?
2. What do you understand about agriculture for nutrition practices?
3. What do you understand about the 3As counselling process for child feeding or agriculture?

Probes:

- 3As steps (assessment, analysis, action)
- Counselling skills (listening and learning, confidence building)
- What remains unclear/questions you still have

1. What do you understand about the men’s and women’s group dialogues?

Probes:

- 7 organizing steps
- Facilitation skills (listening and learning, confidence building)
- What remains unclear/questions you still have

1. What do you understand about your role and responsibilities to implement the SURE programme?

Probes:

- Your role is vis-à-vis the other sector
- Household couselling visits
- Men’s and women’s group dialogues
- Cooking demonstrations
- Gardening demonstrations
- Frequency of services
- Monitoring

1. What do you understand about how to use the SURE tools (job aids, pocket guide, seasonal food calendar?)

Probes:

- Who
- When/frequency
- What remains unclear/questions you still have

1. What do you understand about how to use the SURE monitoring forms?

Probes:

- - Who
  - When/frequency
  - What remains unclear/questions you still have

1. What are your views about integrating your work with the other sector?

Probes:

- - Feasibility
  - Frequency
  - Participation at the coordination committee
  - Monitoring

1. What do you think are the barriers to your implementation of the SURE activities?
