# Supplementary material for: The fidelity and dose of message delivery on infant and young child feeding practice and nutrition sensitive agriculture in Ethiopia: a qualitative study from the Sustainable Undernutrition Reduction in Ethiopia (SURE) programme
Source: J Health Popul Nutr. 2019 Oct 21;38:29. doi: 10.1186/s41043-019-0187-z (PMC6805331; doi:10.1186/s41043-019-0187-z)
Supplement: Supplementary file 3 — Additional file 3. Topic guide for key informant interviews with health and agriculture development agents [file 41043_2019_187_MOESM3_ESM.docx]

## Additional file 3: Topic guide for key informant interviews with health and agriculture development agents

1. What do you understand about infant and young child feeding practices?
2. What do you understand about agriculture for nutrition practices?
3. What do you understand about how to use the SURE tools (job aids?) *Show the job aids.*

Probes:

- Who
- When/frequency
- What remains unclear/questions you still have

1. What do you understand about your role and responsibilities to implement the SURE programme?

Probes:

- Discussions during 1-5
- Community mobilization for group dialogues, cooking demonstrations, gardening demonstrations
- Frequency

1. What are the problems you have when you discuss child feeding and agricultural practices?
2. What are your suggestions to help you improve your work?
