# Supplementary material for: The fidelity and dose of message delivery on infant and young child feeding practice and nutrition sensitive agriculture in Ethiopia: a qualitative study from the Sustainable Undernutrition Reduction in Ethiopia (SURE) programme
Source: J Health Popul Nutr. 2019 Oct 21;38:29. doi: 10.1186/s41043-019-0187-z (PMC6805331; doi:10.1186/s41043-019-0187-z)
Supplement: Supplementary file 4 — Additional file 4. Topic guide for key informant interviews with mother-father pairs in a household [file 41043_2019_187_MOESM4_ESM.docx]

## Additional file 4: Topic guide for key informant interviews with mother-father pairs in a household

1. Please tell us about the visit made by HEWs and AEWs to your household.
2. What did you hear about infant and young child feeding recommendations?

Probe:

- Plan of action agreed?

1. What did you hear about agriculture recommendations?

Probe:

- Plan of action agreed?
- Inputs received (poultry or improved seeds)
- Experience and use of inputs

1. What was your experience of using the job aids?

Probes:

- SURE household card / food group poster
- Seasonal food calendar
- Job aid (books)

1. What do you think is the role of the husband and wife in child feeding and agriculture?
2. What was your experience of attending events in the community:

Probes:

- Cooking demonstration
- Garden demonstration
- Growth monitoring and promotion (weight)
- Acute malnutrition screening (MUAC)

1. What radio messages have you heard about how to feed your infant or young child, or how to produce foods for your infant or young child?
