# Supplementary material for: The fidelity and dose of message delivery on infant and young child feeding practice and nutrition sensitive agriculture in Ethiopia: a qualitative study from the Sustainable Undernutrition Reduction in Ethiopia (SURE) programme
Source: J Health Popul Nutr. 2019 Oct 21;38:29. doi: 10.1186/s41043-019-0187-z (PMC6805331; doi:10.1186/s41043-019-0187-z)
Supplement: Supplementary file 5 — Additional file 5. Topic guide for focus group discussions with health extension workers [file 41043_2019_187_MOESM5_ESM.docx]

## Additional file 5: Topic guide for focus group discussions with health extension workers

1. What was your experience of conducting joint household visits?

Probes:

- Commitment of AEWs
- Frequency of visits
- Participation of both mother and father
- Integrated planning with AEW
- Workload

1. How did you find using the 3 A’s counselling technique to ask, analyse and negotiate actions to improve infant and young child feeding practices?
2. What was your experience of using the job aids during the household visits?
3. How did you find counselling about the role of fathers in child feeding?
4. What was your experience of running women’s group at the community level?
5. What was your experience of running other SURE activities:

Probes:

- Growth monitoring and promotion
- Cooking demonstrations
- Acute malnutrition screening
- Vitamin A and deworming
- Iron folate distribution

1. What was your experience of participating in the kebele multisectoral coordination team?
2. Please describe your experience of receiving supportive supervision or on-the-job training.
3. What do you think are the challenges to the effective implementation of the SURE programme?
