# Supplementary material for: The fidelity and dose of message delivery on infant and young child feeding practice and nutrition sensitive agriculture in Ethiopia: a qualitative study from the Sustainable Undernutrition Reduction in Ethiopia (SURE) programme
Source: J Health Popul Nutr. 2019 Oct 21;38:29. doi: 10.1186/s41043-019-0187-z (PMC6805331; doi:10.1186/s41043-019-0187-z)
Supplement: Supplementary file 6 — Additional file 6. Topic guide for focus group discussions with agriculture extension workers. [file 41043_2019_187_MOESM6_ESM.docx]

## Additional file 6: Topic guide for focus group discussions with agriculture extension workers

1. What was your experience of conducting joint household visits?

Probes:

- Commitment of HEWs
- Frequency of visits
- Participation of both mother and father
- Integrated planning with HEW
- Workload

1. How did you find using the 3 A’s counselling technique to ask, analyse and negotiate actions to improve nutrition-sensitive agriculture practices?
2. What was your experience of using the job aids, seasonal food calendar and food group poster during the household visits?
3. How did you find counselling about the role of mothers in agriculture?
4. What was your experience of running men’s groups at the community level?
5. What was your experience of running other SURE activities:

Probes:

- Gardening demonstrations
- Cooking demonstrations
- Supporting use of poultry or improved seed inputs

1. Please describe the household selection process to receive agriculture inputs.

Probes:

- Poultry
- Improved seeds

1. What was your experience of participating in the kebele multisectoral coordination team?
2. Please describe your experience of receiving supportive supervision or on-the-job training.
3. What do you think are the challenges to the effective implementation of the SURE programme?
