# Supplementary material for: The fidelity and dose of message delivery on infant and young child feeding practice and nutrition sensitive agriculture in Ethiopia: a qualitative study from the Sustainable Undernutrition Reduction in Ethiopia (SURE) programme
Source: J Health Popul Nutr. 2019 Oct 21;38:29. doi: 10.1186/s41043-019-0187-z (PMC6805331; doi:10.1186/s41043-019-0187-z)
Supplement: Supplementary file 7 — Additional file 7. Observation note-taking form [file 41043_2019_187_MOESM7_ESM.docx]

## Additional file 7: Observation note taking form

Date: ___________________ Starting time: ___________

Region: _____________ Woreda: ______________ Kebele: __________ Place: ___________

Event observed (please circle):

***SURE MCT meeting SURE HH visit Cooking demonstration Farmers demonstration***

***SURE Men’s group dialogue SURE Women’s group dialogue***

Participants of the event (please describe what kind of people participated the event):

_______________________________________________________

Number of participants in the event: ______________________

Your planned observation agenda: ____________________________________________________________________________________________________________________________________________________

Overview of what you observed (summary of proceedings, topics/sessions, context, how the demonstration was introduced, steps followed during the demonstration, materials used for demonstration, active participation of attendants etc.)

__________________________________________________________________________________________________________________________________________________________________________________________________________________________________________________________________________________________________________________________________________________

___________________________________________________________________________

Observation ending time: _________________

Name of the observer: ____________________

Signature: ____________________________
